# Supplementary material for: Electrophoresis-Correlative Ion Mobility Deepens Single-Cell Proteomics in Capillary Electrophoresis Mass Spectrometry
Source: Mol Cell Proteomics. 2024 Dec 19;24(2):100892. doi: 10.1016/j.mcpro.2024.100892 (PMC11875174; doi:10.1016/j.mcpro.2024.100892)
Supplement: Supplemental Figs. S1–S3 [file mmc1.pdf]

Supporting Information

Electrophoresis-Correlative Ion Mobility Deepens Single-cell Proteomics in Capillary Electrophoresis Mass Spectrometry

Bowen Shen[1], Fei Zhou[1], and Peter Nemes[1]*

[1]Department of Chemistry & Biochemistry, University of Maryland, College Park, MD 20742

***Correspondence to:** Department of Chemistry & Biochemistry, University of Maryland, College Park, 8051 Regents Drive, College Park, MD 20742, USA. Phone: (1) 301-405-0373. Fax: (1) 301-314-9121. E-mail: nemes@umd.edu.

TABLE OF CONTENTS

FIGURES 2

Figure S1 2

Figure S2 3

Figure S3 4

FIGURES

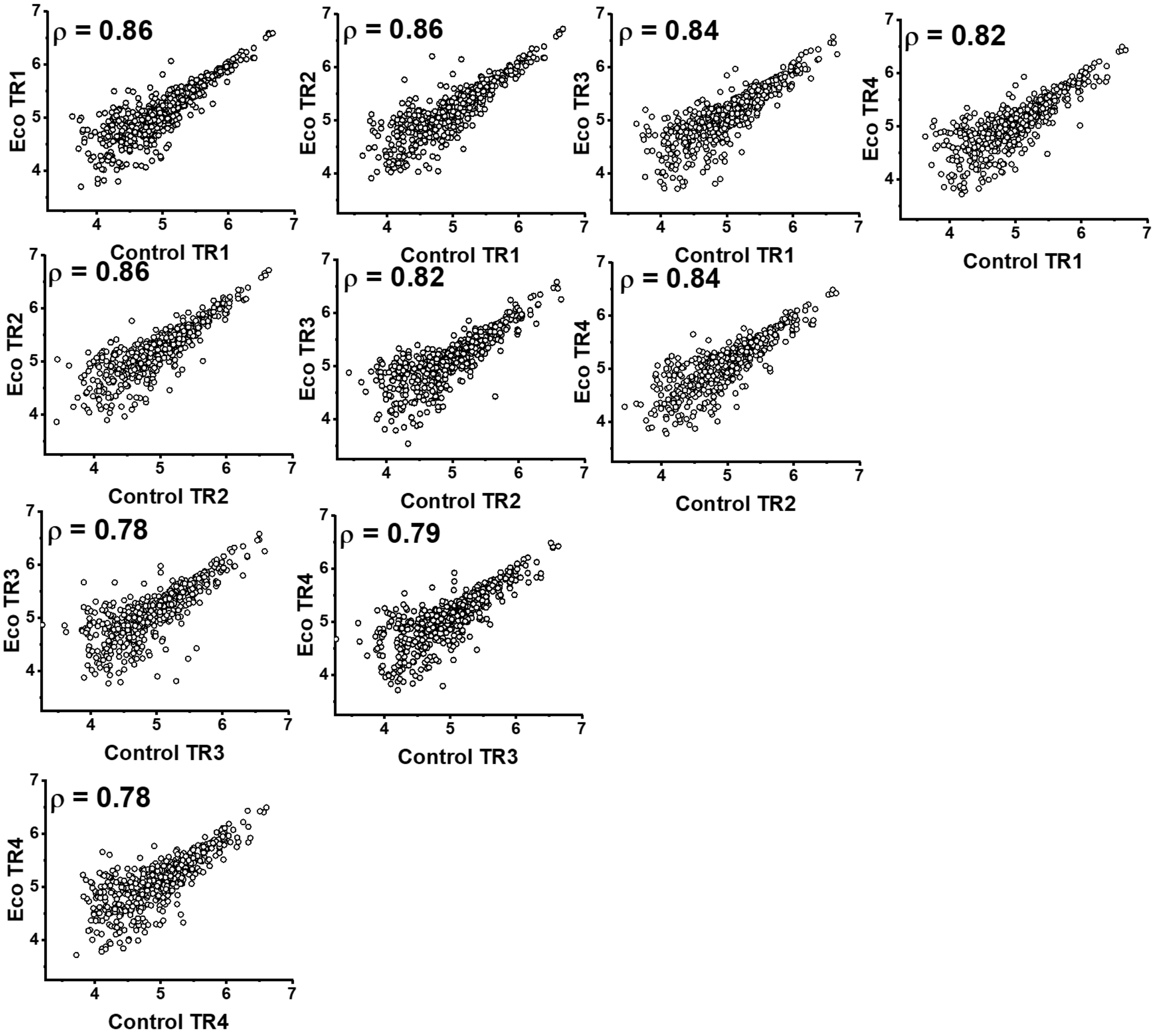

Figure S1. Benchmarking of Eco-IMS proteome quantification vs. the classical approach (Control). Ca. 500 pg was measured from the HeLa proteome digest in 4 technical replicates (TR) using each method. Protein concentrations were estimated based on $\log_{10}$-transformed and median-normalized label-free quantification indexes (MSFragger), without using match-between-runs. These values are plotted on the dependent and independent axes to assess reproducibility. We considered Pearson correlation moments of $\rho = 0.78–0.86$ to be sufficient indication of good quantitative fidelity.

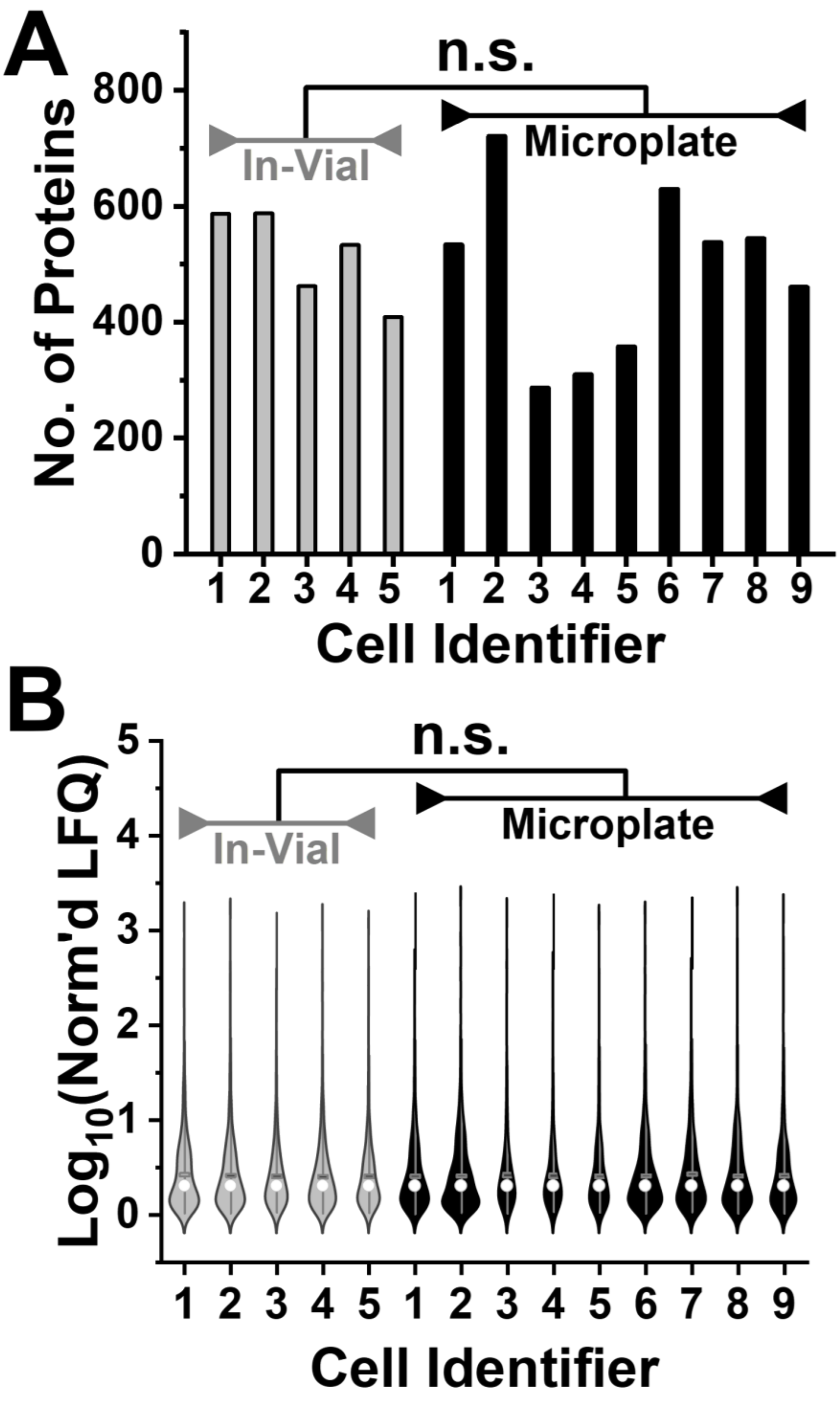

Figure S2. Comparison of **(A)** protein identification and **(B)** quantification performance with the sample processing between the classical plastic vials vs. the fluorosilane-coated microplates. The microdroplet format allowed us to digest single-cell proteomes using limited reagents ca. 5-times faster in similar performance between the approaches ($p = 0.70$). The LFQ data were median normalized and $\log_{10}$ transformed. Key: n.s., not significant (**Fig. S2A**, unpaired student t-test; **Fig. S2B**, Kruskal-Wallis analysis).

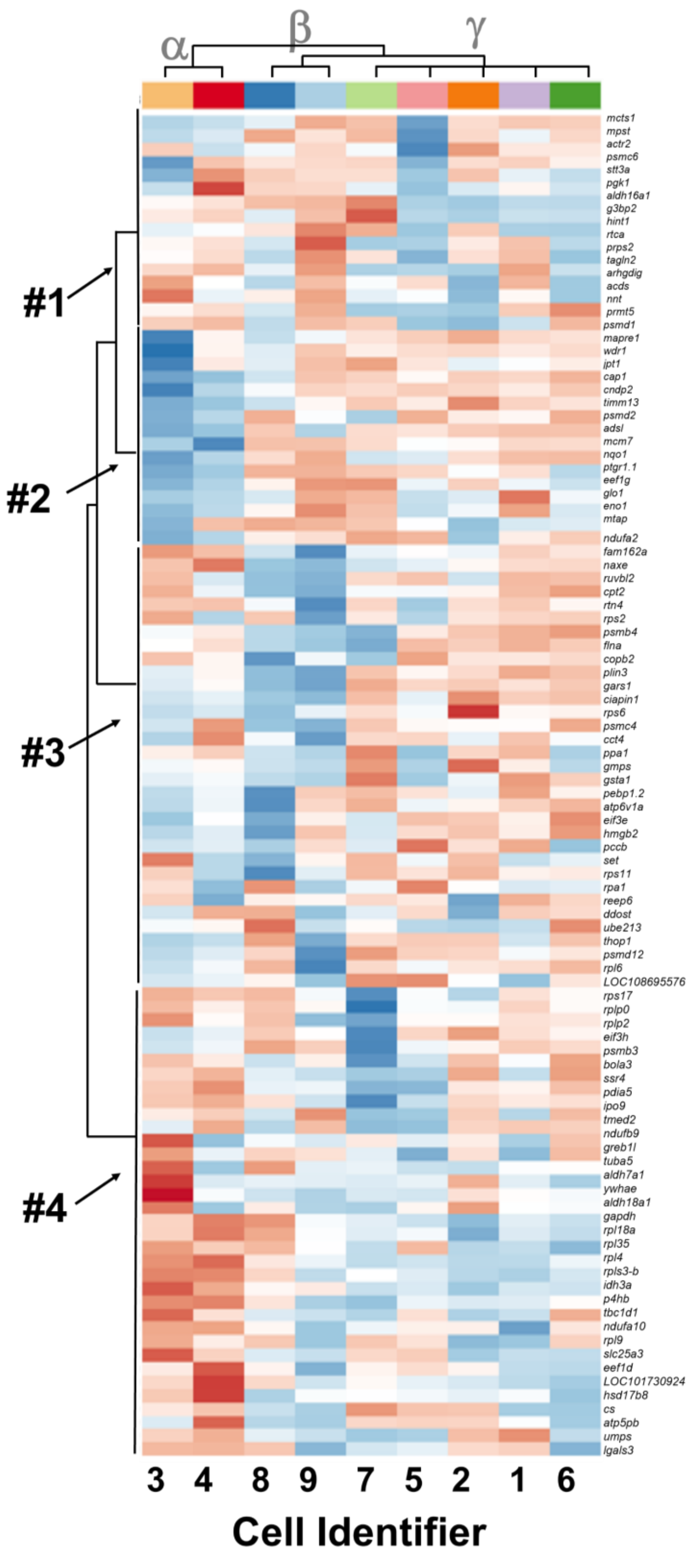

Figure S3. Close-up of the hierarchical cluster analysis (HCA)–heatmap (**Fig. 5D**). The 100 most significantly differently abundant proteins are shown. Each protein is labeled by the name of the corresponding gene following the *Xenopus* nomenclature.
